# Identification of a novel de novo pathogenic variant in *GFAP* in an Iranian family with Alexander disease by whole-exome sequencing

**DOI:** 10.1186/s40001-022-00799-5

**Published:** 2022-09-10

**Authors:** Katayoun Heshmatzad, Niloofar Naderi, Tannaz Masoumi, Hamidreza Pouraliakbar, Samira Kalayinia

**Affiliations:** 1 Cardiogenetic Research Center, Rajaie Cardiovascular Medical and Research Center, Iran University of Medical Sciences, Tehran, Iran; 2 Cardiovascular Imaging Research Center, Rajaie Cardiovascular Medical and Research Center, Iran University of Medical Sciences, Tehran, Iran

**Keywords:** Infantile Alexander disease, *GFAP*, Leukodystrophy, Whole-exome sequencing, Genetics, In silico analysis

## Abstract

**Background:**

Alexander disease (AxD) is a rare leukodystrophy with an autosomal dominant inheritance mode. Variants in *GFAP* lead to this disorder and it is classified into three distinguishable subgroups: infantile, juvenile, and adult-onset types.

**Objective:**

The aim of this study is to report a novel variant causing AxD and collect all the associated variants with juvenile and adult-onset as well.

**Methods:**

We report a 2-year-old female with infantile AxD. All relevant clinical and genetic data were evaluated. Search strategy for all AxD types was performed on PubMed. The extracted data include total recruited patients, number of patients carrying a *GFAP* variant, nucleotide and protein change, zygosity and all the clinical symptoms.

**Results:**

A novel de novo variant c.217A > G: p. Met73Val was found in our case by whole-exome sequencing. In silico analysis categorized this variant as pathogenic. Totally 377 patients clinically diagnosed with juvenile or adult-onset forms were recruited in these articles, among them 212 patients were affected with juvenile or adult-onset form carrier of an alteration in *GFAP*. A total of 98 variants were collected. Among these variants c.262C > T 11/212 (5.18%), c.1246C > T 9/212 (4.24%), c.827G > T 8/212 (3.77%), c.232G > A 6/212 (2.83%) account for the majority of reported variants.

**Conclusion:**

This study highlighted the role of genetic in AxD diagnosing. It also helps to provide more information in order to expand the genetic spectrum of Iranian patients with AxD. Our literature review is beneficial in defining a better genotype–phenotype correlation of AxD disorder.

## Introduction

Alexander disease (AxD) (OMIM #203450) is a rare leukodystrophy first described in 1949 with usually infantile manifestation. The exact prevalence of AxD is not known, however a Japanese investigation estimated an incidence of 1 person in 2.7 million. This disorder belongs to a group of neurological diseases denoted as leukodystrophies affecting the central nervous system (CNS) white matter and characterized by myelin sheath defects or abnormal development of myelin sheath [1, 2]. According to age of onset, AxD is classified in to three subgroups naming infantile, juvenile and adult forms [3]. Patients affected with infantile AxD present various symptoms such as seizures, megalencephaly, developmental delay, progressive deterioration and increased neonatal patients severity within first two years after birth [4]. Juvenile form with the age of onset (2–14 years of age) is characterized by symptoms including ataxia, hyperreflexia, bulbar symptoms. Juvenile form has milder progression and preserved cognitive and motor function comparing to infantile form. Adult AxD patients have more similarities to the juvenile form and manifest mainly spastic paraparesis, palatal myoclonus, bulbar symptoms and ataxia [5]. AxD is usually diagnosed based on the results of CT and MRI characteristic appearances—reference. Frontal predominance involvement, hindbrain involvement, medulla oblongata and cervical spinal cord atrophy are indicators of younger patients and patients with later onset, respectively [6–8]. This autosomal dominant disorder is usually the consequence of defects in *GFAP* gene [9]. Sporadic cases should be mentioned briefly *GFAP* is located within chromosome 17q21 consists of nine exons spreading 9.8 kb length encoding a 432 amino acid protein. This protein belongs to intermediate filament proteins and has considerable and key roles in astrocytes morphology and motility regulation and astrocytes and oligodendrocytes interaction. The exact and precise mechanism through which GFAP function is not completely understood, however, it is believed that gain of function mutations in *GFAP* affects and disrupts intermediate filaments dimerization leading to abnormal aggregation of proteins and cytoskeleton collapse [3, 10, 11]. *GFAP* identification and sequencing have increased the level of diagnosis accuracy and statistical analysis have evaluated the relationships between onset age and the *GFAP* genotype and its clinical outcomes [12]. Nearly all of the *GFAP* disease-causing mutations are heterozygous single base-pair alterations located in the coding region especially in central rod domain conserved α-helices. The remaining mutations are near the N-terminus precoil domain and C-terminal tail domain [3, 13]. In this study, we report a *GFAP* novel variant in a 2-year-old female affected with infantile form and conduct a comprehensive review on all of the reported *GFAP* mutations in patients with adult and juvenile forms as well.

## Methods

### Case clinical features and demographic data

**Fig. 1:**
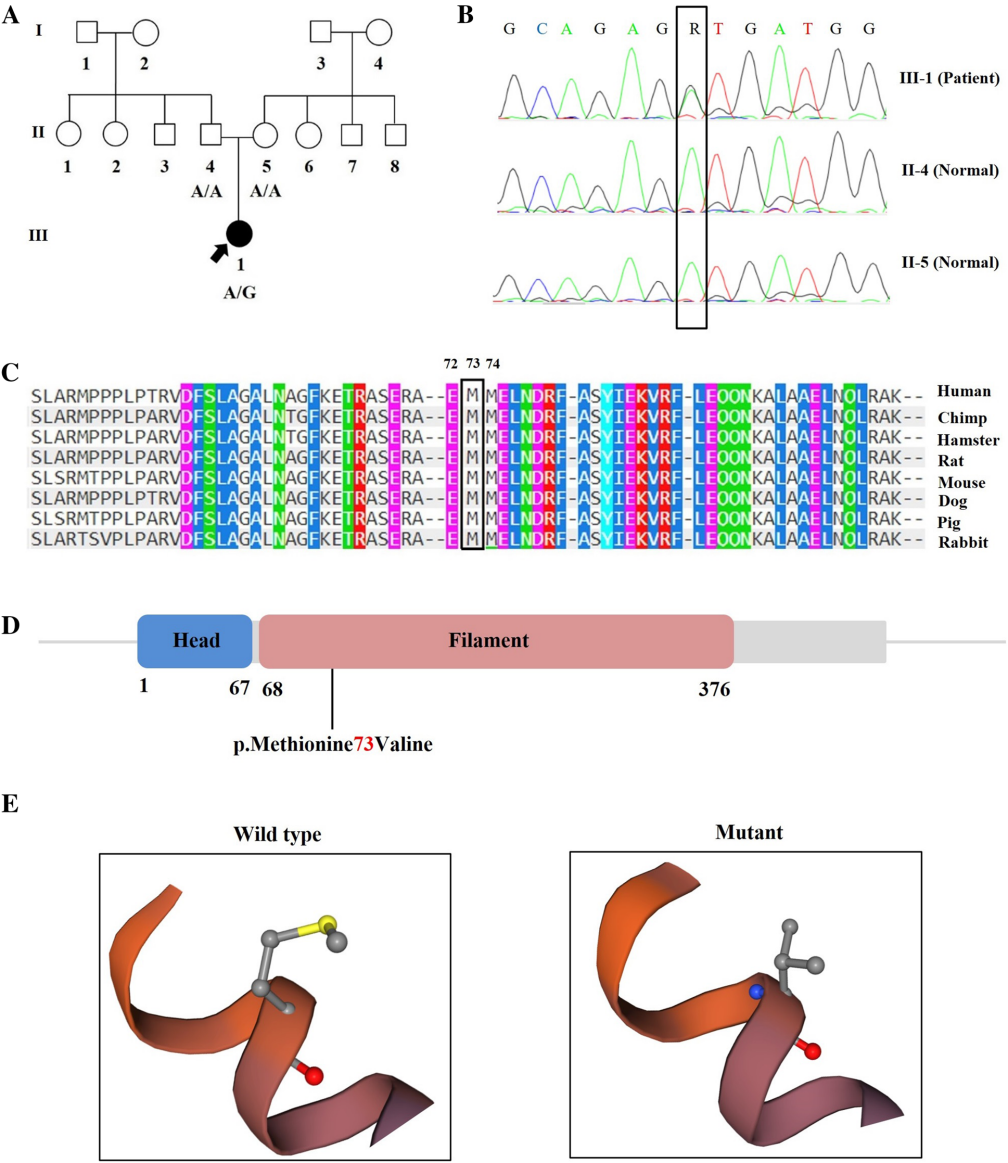
Genetic and protein changes of GFAP. **A** The pedigree of a family with Alexander disease. The black arrow indicates proband. Affected and unaffected individuals are represented by filled and clean symbols, respectively. **B** Sanger sequencing results show that a novel de novo variant in the *GFAP* was found in the proband (III-1) and normal sequence of her parents (II-4/II-5). **C** Conservation of p.Met73Val variant across various species has been shown. The variant site is highly conserved in various species. **D**, **E** Schematic view of GFAP and the position of mutation p.Met73Val

A 2-year-old female patient referred to Cardiogenetic Research Center, Rajaie Cardiovascular Medical and Research Center, Iran University of Medical Sciences, Tehran, Iran, suffering from developmental delay and vomiting during one year after her birth. She was born through cesarean delivery and she was the only child of one healthy non-consanguineous parents (Fig. 1A). Her birth weight and head circumference were 2350 g and 33.9 cm, respectively. At age 24 months, she manifested some further symptoms including seizure and motor and speech delays. She could not also sit independently. The patient presented spasticity and increased deep tendon reflexes (DTRs). Further neurological examination also revealed ataxia and she had also gait disturbance. The clinical surveys of other available members of the pedigree were normal. After conducting clinical evaluations and family history recording and genetic counselling, whole-exome sequencing [14] was conducted for precise diagnosis. Identified candidate variant was confirmed and segregated in family members using PCR and direct Sanger sequencing. The study was performed in accordance with the Helsinki Declaration and has been approved by the Rajaei Cardiovascular, Medical, and Research Center ethics committee (IR.RHC.REC.1400.077).

#### MRI

Her first brain magnetic resonance imaging (MRI) at the age of 24 months indicated diffuse hyperintensity in periventricular and subcortical white matter of frontal and parietal lobes. Furthermore, basal ganglia indicated hyperintensity on apparent diffusion coefficient (ADC) maps. The brainstem and cerebellum had no abnormalities. Her MRI suggested leukodystrophy or hypoxic–ischemic encephalopathy. Her MRI reveals white matter involvement.

### Whole-exome sequencing

Informed consent was obtained from the proband’s parents. DNA extraction was conducted according to salting out method. The quality and quantity of extracted DNA was checked by agarose gel electrophoresis and NanoDrop (Thermo Fisher Scientific, USA). DNA sample of the proband (III-1) (Fig. 1A) was subjected to WES and was conducted using at Macrogen (Seoul, South Korea) and raw data (fastq) was analyzed by Cardiogenetic Research Center, Rajaie Cardiovascular, Medical, and Research Center, Tehran, Iran.

The short reads alignment with human reference genome (UCSC build37/hg19) was performed by BWA (http://bio-bwa.sourceforge.net/) [15]. Any alterations including insertions/deletions (indels), single-nucleotide polymorphisms (SNPs) and polymerase chain reaction (PCR) duplicates removal were detected using Picard (http://picard.sourceforge.net/), SAMtools (http://www.htslib.org/) [16], and GATK (https://www.broadinstitute.org/gatk/) [17]. After annotation by annovar (http://annovar.openbioinformatics.org) [18], variants with minor allele frequency (MAF) < 0.05 were selected and filtered. In order to assess deleterious effects of variants, bioinformatics tools were applied including combined annotation dependent depletion (CADD; https://cadd.gs.washington.edu/home) [19], sorting intolerant from tolerant (SIFT; https://sift.bii.a-star.edu.sg/) [20], MutationTaster (http://www.mutationtaster.org/) [21], protein variation effect analyzer (PROVEAN; http://provean.jcvi.org/index.php) [22], polymorphism phenotyping v2 (PolyPhen-2; http://genetics.bwh.harvard.edu/pph2/) [23], genomic evolutionary rate profiling (GERP; http://mendel.stanford.edu/SidowLab/downloads/gerp/), and CLUSTALW (https://www.genome.jp/tools-bin/clustalw).

### Validation, and bioinformatics analysis

The validation of identified variant was confirmed in the proband and segregated in other family members by PCR and direct Sanger-sequencing. PCR was performed using specific primers (forward primer: TTCATAAAGCCCTCGCATC, reverse primer: CGCTTCCAACTCCTCCTTTA) on a SimpliAmp Thermal Cycler (Thermo Fisher Scientific) and products were sequenced on an ABI Sequencer 3500XL PE (Applied Biosystems). The sequences were analyzed by CodonCode Aligner 7.1.2 (https://www.codoncode.com/aligner/).

### Search strategy and data extraction

The combination of following keywords GFAP and Alexander disease, “GFAP mutations” and GFAP” [title/abstract] were used searching PubMed. Totally 954 articles were collected and after duplicate removal, 868 articles remained. The inclusion criteria include patients affected with juvenile and adult-onset form of AxD who carried an alteration in *GFAP*.

According to our defined inclusion criteria, nucleotide and protein change, zygosity, number of total recruited patients and *GFAP* carriers, main clinical symptoms were extracted from the selected articles (Table 1). All the collected variants were analyzed by different in silico tools such as Clinvar, SIFT, Mutation Taster, PROVEAN, GERP, ACMG, CADD and Polyphen-2 (Table 2).

**Table 1:** Data extraction

No.	Mutation	Protein change	Total recruited patients	Number of carriers	Age	DTR	Ataxia	Hypertonia	Myoclonus	Encephalopathy	Scoliosis	Bulbar signs	Nystagmus	Palatal myoclonus	Spasticity	Status epilepticus	Seizures	Atrophy	Mental retardation	Developmental Delay	Gait	Macrocephaly	Slurred speech	Clumsiness	Unsteadiness	Elective mutism	Standing on one foot	Other	Ref.
1	c.214G > A c.1235C > T	E72K T412I	2	2	26 33							2	2								2		1						[33]
2	c.731C > T	A244V	65	1	10											1	1		1		1	1	1	1	1	1	1		[39]
3	c.250G > A	R79H	1	1	6												1			1			1						[40]
4	c.988C > G c.994G > A	p. Arg330Gly p.Glu332Lys	1	4	57 28 53 64							2		1	2			4			2		1						[41]
5	c.868C > G c.729C > T	p.Q290E p.R239C	3	1 1	14 10	1			1	1	2	2	1				1												[42]
6	c.1157A > G c.1127G > A	p.Asn386Ser p.Arg376Gln		2	53 59		1					1											1						[43]
7	c.235C > T c.*29C > T	p.R79C	1		10	B					1	1			1		1			1									[44]
8	c.628G > A	E312K						1				1					1			1									[45]
9	c.250G > A	R79H	11	1	10																								[3]
10	c.934G > T	E312ter	1		67																							SM	[46]
11	681G > C	E223Q		1	40		1					1																	[47]
12	c.382G > A	D128N	1	1	65																							RF C M	[48]
13	c.236G > C c.1246C > T c.1076T > C c.209G > A c.208C > T	R79P R416W L359P R70Q R70W	13	5	5 13 19 35 43		2					4			1		1												[27]
14	c.53G > T	p.Gly18Val	1	1	46										1														[49]
15	c.382 G > A	p.Asp128Asn	1	1	52																							RF P	[50]
16	c.219G > C	p.M73I	1	1	49		1			1	1				1													H	[30]
17	c.809G[C	p.Arg270Pro	1	1	36		1					1	1																[51]
18	c.1245G > A	M451I	3	3	38 35 60				1		2		1										3					M (3)	[52]
19	c.1076T > C c.1178G > T c.1246C > T c.209G > A c.613G > A c.208C > T c.994G > A c.613G > A c.1193C > A c.382G > A	p.L359P p.S393I p.R416W p.R70Q p.E205K p.R70W p.E332K p.E205K p.S398Y p.D128N	11	10	26 36 26 39 30 43 61 58 52 64		6				1	5						10											[53]
20	c.619-C > G	NA	3	1	39							1						1										M	[54]

No.	Mutation	Protein change	Total recruited patients	Number of carriers	Age	DTR	Ataxia	Hypertonia	Myoclonus	Encephalopathy	Scoliosis	Bulbar signs	Nystagmus	Palatal myoclonus	Spasticity	Status epilepticus	Seizures	Atrophy	Mental retardation	Developmental Delay	Gait	Macrocephaly	Slurred speech	Clumsiness	Unsteadiness	Elective mutism	Standing on one foot	Other	Ref
21	c.197G > A*(Homo)	p.Arg66Gln	1	1	16	I	1		1			1	1																[35]
22	c.620A > T	p.Glu207Val	1	1	52		1					1																	[55]
23	c.232G > C c.276C > T c.276C > T c.1260C > T c.1260C > T	D78H R88C R88C R416W R416W	13	5	8 9 10 11 12							3					1												[28]
24	c.219G > T	M73I	1	1	48							1																H	[31]
25	c.1289G > A c.1289G > A c.1290C > A	p.Arg430His p.Arg430His p. Arg430Arg	3	3	42 45 8		2					3	2		1		1												[56]
26	c.197G > A	R66Q	1	1	54							1			1			1					1						[57]
27	c.799G > C 128C > G	A267P -	1	1	25	B			1		1	1			1						1							IM	[58]
28	c.770AG	Y257C	1	1	59								1								1							M	[59]
29	c.1079A > T	D360V	1	1	9										1						1		1					Mt	[60]
30	c.1177A > C	S393R	1	1	50	I							1		1			1										RF	[61]
31	3 bp deletion	-	1	1	8				1			1									1								[37]
32	c.302T > C	L101P	1	1	26		1					1	1		1			1			1							IM	[62]
33	c.1289G > A	p.Arg430His	1	1	32	B						1			1			1											[63]
34	c.1246C > T	p.Arg416Trp	1	1			1		1						1			1				1							[64]
35	c.262C > T c.278A > C c.628G > A	R88C Q93P E210K	3	3	9 10 24						1	2			2						1							M (2)	[65]
36	c.382G > A	p.Asp128Asn	1	1	68	I	1					1	1								1								[66]
37	c.1246C > T	p.R416W	1	1	28		1				1	1			1														[67]
38	c.368T > C	p.Leu123Pro	1	1	51							1			1			1			1								[68]
39	c.250 G > A	p.Arg79His	1	1	21	I							1					1			1		1						[69]
40	c.739T > C c.1250A > C c.1277A > T	p.Ser247Pro p.Asp417Ala p.Gln426Leu	3	3	26 32 46																1								[70]
41	c.262C > T	p.Arg88Cys	6	1								1																	[71]
42	c.934G > T	p.(E312*)	1	1	67																								[72]
43	c.1087A > G	p.Ile363Val	1	1	3		1						1				1	1											[73]
44	c.827G > T	R276L	3	3	33	I						1			1														[6]
45	c.827G > T	p.R276L	1	1	11						1						1												[74]

No.	Mutation	Protein change	Total recruited patients	Number of carriers	Age	DTR	Ataxia	Hypertonia	Myoclonus	Encephalopathy	Scoliosis	Bulbar signs	Nystagmus	Palatal myoclonus	Spasticity	Status epilepticus	Seizures	Atrophy	Mental retardation	Developmental Delay	Gait	Macrocephaly	Slurred speech	Clumsiness	Unsteadiness	Elective mutism	Standing on one foot	Other	Ref
46	c.827G > T	p.R276L	1	1	57							1	1		1														[75]
47	c.1070C > T	L357P	1	1	7								1																[76]
48	c.262C > T	R88C	11	1	29												1					1							[77]
49	c.617A > C	Glu206Ala	1	1	40		1					1			1						1								[78]
50	c.724T > A c.724T > A	pY242N pY242N	2	2	37 38		2					2	1					2											[79]
51	c.221T > C	M74T	1	1	50	I						1						1											[80]
52	c.613G > A c.613G > A c.1193C > A c.382G > A c.1076T > C c.1178G > T c.1246C > T c.209G > A c.208C > T c.994G > A	p.E205K p.E205K p.S398Y p.D128N p.L359P p.S393I p.R416W p.R70Q p.R70W p.E332K	11	10	30 54 52 62 19 33 13 34 30 43		6		3		2	4	5		4						8								[81]
53	c. 211G > A	R66Q	30	1	40					1		1					1												[12]
54	c1178G > T	S393I	1	1	35							1			1														[82]
55	c.1100G > C	E362D	1	1	13												1						1						[83]
56	c.365_373dup	p.Arg124_Leu125insGlnLeuAr	1	1	32							1			1													C	[84]
57	c. 1148C > T	T383I	1	1	55	B															1		1						[85]
58	1006T > C	L331P	1	1	7																								[86]
59	c.778A > C	p.Lys260Gln	1	1	25							1			1													C	[87]
60	c.262C > T c.262C > T c.262C > T c.1119G > C c.236G > T c.226C > T c.262C > A c.262C > T c.236G > A c.235C > T c.231T > A c.235C > T c.236G > A c.716G > A c.235C > T	p.R88C p.R88C p.R88C p.E373D p.R79L p.L76F p.R88S p.R88C p.R79H p.R79C p.N77K p.R79C p.R79H p.R239H p.R79C	22	15													9			4								M(3)	[88]
61	c.1157A > G	p.N386S	1	1	72	I												1											[89]
62	c.803C > A c.1246C > T c.1157A > G c.1157A > G c.731C > T c.306C > A c.724T > A c.724T > A c.1246C > T c.1070C > T c.372_373insGAA c.368T > C	A268D R416W N386S N386S A244V G301D N102K Y242N Y242N R126_L127dup R416W L357P R124_L125insE L123P	19		30 38 63 59 32 51 66 40 35 22 45 18 46 45		14	8				13			14														[90]
63	c.262C > T	R88C	1	1	7										1		1					1						M	[91]
64	c.726_728dupAGG	p.E243dup	1	1	22		1					1	1								1		1						[92]
65	c.232G > A c.232G > A c.232G > A	D78N D78N D78N	3	3	64 55 32	I(2)	1				1	2	1								1		2					H	[93]
66	c.611A > G	p.His204Arg	1	1	55		1	1				1									1							H	[94]
67	c.1246C > T	R416W	1	1	7						1	1																C	[95]
68	C1260T	R416W	1	1	12						1										1								[96]
69	c.273G > C	p.V87L	1	1	31	I	1					1	1		1		1	1			1							H	[97]
70	c.273G > C c.273G > C c.715C > T c.208C > T c.236G > A c.221T > C c.1070C > T	V87G V87G R258C R70W R79H M74T L357P	12	7	44 33 59 64 36 51 18	I5	3		3			5	3					6											[98]
71	c.236G > A c.236G > T c.262C > T c.715C > T c.715C > T c.731C > T c.1079A > T c.1119G > C c.221T > C c.302T > C c.731C > T c.773G > C c.791T > C c.1090G > A c.1090G > A c.827G > T(3 c.1126C > T c.1178G > T c.1193C > T c.273G > C	R79H R79L R88C R239C R239C A244V D360V E373D M74T L101P A244V L258C L264P A364T A364T R276L(3) R376W S393I S398F V87G	31	31			7					18	11										4						[2]
72	c.619G > A c.704T > C c.704T > C c.187A > C c.731C > T c.619G > C c.715C > T	Glu207Lys Leu235Pro Leu235Pro Lys63Gln Ala244Val Glu207Gln Arg239Cys	10	7	10 3 3 21 9 10 4		2				1	5			4						3								[8]
73	c.234C > G	D78E	1	1									1																[99]
74	c.1158C > A	N386K	1	1	79		1														1								[100]
75	c.208C > T	p.Arg70Trp	1	1	38		1					1	1																[101]
76	c.380_385dupGCGGCT c.256_259delinsGAGT c.262C > T c.262C > T c.1246C > T c.628G > A c.262C > T	p.Arg126_Leu127dup p.Lys86_Val87delinsGluPhe p.Arg88Cys p.Arg88Cys p.Arg416Trp p.Glu210Lys p.Arg88Cys	7	7	2		1					1					1				2	1							[7]
77	c.469G > A c.1245G > A	D157N M4151	1	1	13		1					1									1								[102]
78	c.274T > G(3)	G87V	3	3	53 27 32	I3						1						3			2		3						[103]
79	c.1154 C > G IVS4-24_812 c.259 G > A c.715C > G c.701C > A c.1154 C > G c.209 G > A c.1118A > C	p.Ser385Cys NA p.Val87lle P.Arg239Gly p.Ala234Asp p.Ser385Cys p.Arg70Gln p.Glu373Ala	13	8	23 38 12 27 13 44 39 33		3					4	3		5						6								[104]
80	c.692T > A	p.Leu231His	1	1	50		1					1									1								[105]
81	c.988C > G c.994G > A	p.R330G p.E332K	1	1	56																								[106]
82	c.236G > A	p.R79H	1	1	36		1								1				1		1								[107]
83	c.236G > A	R79H	1	1	38																								[108]
84	c.232G > A(3)	p.D78N	1	3		I(2)	1				1	2	1								1		1						[93]
85	c.221T > C	p.M74T	1	1	56								1		1														[109]
86	c.1157A > G c.628G > A c.716G > A c.208C > T	N386S E210K R258H R70W	4	4	62 58 60 64		4					4	3		4														[110]

**Table 2:** Bioinformatics analysis of *GFAP* collected variants related to Alexander disease

No.	Position on Chromosome 17 (GRCh37)	HGVS DNA	HGVS protein	Exon/intron	SNP ID	Transcript	Coil	ClinVar	SIFT	MutationTaster	PROVEAN	FATHMM	GERP	ACMG	CADD	PolyPhen-2
1	42987997	c.1157A > G	p.Asn386Ser	E	rs61726471	ENST00000253408	Tail	-	T	DC	N	D	5.13	LP	17.83	B
2	42992647	c.208C > T	p.Arg70Trp	E	rs60343255	ENST00000253408	Head	P	D	DC/P	D	D	4.82	P	24.1	PD
3	42992549	c.306C > A	p.Asn102Lys	E	-	ENST00000586793.1	Coil1A	-	T	DC	N	T/D	4.69	LP	21.8	PD
4	42988006	c.1148C > T	p.Thr383Ile	E	rs267607517	ENST00000586793.1	Tail	P	D	DC/P	D	D	5.13	LP	25.4	PD
5	42992644	c.211G > A	p.Ala71Thr	E	rs267607522	ENST00000586793.1	Head	NP	D	DC/P	N	D	4.82	LP	23.1	PD
6	42984686	c.*29C > T	NA	3UTR	rs370608748	ENST00000588735.1	-	-	-	-	-	-	5.07	B	-	-
7	42988655	c.1076 T > C	p.Leu359Pro	E	rs267607511	ENST00000586793.1	Coil2B	P	D	DC	D	D	4.25	P	30	PD
8	42988652	c.1079A > T	p.Asp360Val	E	rs62636501	ENST00000586793.1	Coil2B	P	D	DC	D	D	4.25	LP	32	PD
9	42988644	c.1087A > G	p.Ile363Val	E	-	ENST00000586793.1	Coil2B	-	D	DC	N	D	4.25	LP	27.3	PD
10	42988641	c.1090G > A	p.Ala364Thr	E	rs58645997	ENST00000586793.1	Coil2B	P	D	DC	D	D	4.25	P	28.8	PD
11	42988631	c.1100G > C	p.Arg367Thr	E		ENST00000586793.1	Coil2B	-	D	DC	D	D	4.25	P	28.8	PD
12	42988613	c.1118A > C	p.Glu373Ala	E	rs797044589	ENST00000586793.1	Coil2B	P	D	DC	D	D	4.25	P	31	PD
13	42988612	c.1119G > C	p.Glu373Asp	E	-	ENST00000586793.1	Coil2B	-	D	DC	D	D	4.25	P	25.6	PD
14	42988605	c.1126C > T	p.Arg376Trp	E	rs267607512	ENST00000586793.1	Coil2B	P	D	DC	D	D	4.25	P	29.7	PD
15	42988604	c.1127G > A	p.Arg376Gln	E	-	ENST00000586793.1	Coil2B	-	D	DC	D	D	4.25	P	36	PD
16	42988000	c.1154C > G	p.Ser385Cys	E	rs797044590	ENST00000586793.1	Tail	LP/P	D	DC	D	D	5.13	P	28.2	PD
17	42987997	c.1157A > G	p.Asn386Ser	E	rs61726471	ENST00000586793.1	Tail	-	T	DC	N	D	5.13	LP	17.83	B
18	42987996	c.1158C > A	p.Asn386Lys	E	-	ENST00000586793.1	Tail	-	D	DC	N	D	5.13	LP	24.9	B
19	42985512	c.1177A > C	p.Ser393Arg	E	-	ENST00000253408.5	Tail	-	T	DC	N	-	5.23	LP	22.6	PD
20	42985511	c.1178G > T	p.Ser393Ile	E	rs62635764	ENST00000253408.5	Tail	P	T	DC	N	-	5.23	LP	21.9	B

No.	Position on Chromosome 17 (GRCh37)	HGVS DNA	HGVS protein	Exon/intron	SNP ID	Transcript	Coil	ClinVar	SIFT	MutationTaster	PROVEAN	FATHMM	GERP	ACMG	CADD	PolyPhen-2
21	42985496	c.1193C > A	p.Ser398Tyr	E	rs267607508	ENST00000253408.5	Tail	P	P	DC	N	-	5.23	LP	22.4	PD
22	42985496	c.1193C > T	p.Ser398Phe	E	rs267607508	ENST00000253408.5	Tail	P	D	DC	N	-	5.23	LP	22.7	PD
23	42985454	c.1235C > T	p.Thr412Ile	E	rs1597853099	ENST00000253408.5	Tail	LP	D	DC	D	-	5.13	LP	22.4	PD
24	42985444	c.1245G > A	p.Met415Ile	E	-	ENST00000253408.5	Tail	-	D	P	N	-	5.13	VUS/P	21.8	B
25	42985443	c.1246C > T	p.Arg416Trp	E	rs121909717	ENST00000253408.5	Tail	P	D	DC	D	-	5.13	P	21.2	PD
26	42985439	c.1250A > C	p.Asp417Ala	E	rs267607520	ENST00000253408.5	Tail	P	D	DC	D	-	5.13	LP	22.5	B
27	42984754	c.1260C > T	p.Val420 =	E	rs779643685	ENST00000253408.5	Tail	-	-	DC	-	-	4.80	LB	18.95	-
28	42984737	c.1277A > T	p.Gln426Leu	E	rs267607521	ENST00000253408.5	Tail	P	D	DC	D	-	5.34	LP	18.64	PD
29	42987511	c.1289G > A	p.Arg430His	E	rs748860341	ENST00000435360.2	Tail	LP	D	DC/P	D	D	4.78	LP	15.13	PD
30	42987510	c.1290C > A	p.Arg430 =	E	rs775524073	ENST00000435360.2	Tail	LP	D	P	-	-	4.78	VUS/P	11.06	-
31	42992668	c.187A > C	p.Lys63Gln	E	rs60095124	ENST00000586793.1	Head	P	D	DC/P	N	D	4.82	LP	23.5	B
32	42992658	c.197G > A	p.Arg66Gln	E	rs797044569	ENST00000586793.1	Head	Conflict	D	DC	D	D	5.89	LP	29	PD
33	42992647	c.208C > T	p.Arg70Trp	E	rs60343255	ENST00000586793.1	Head	P	D	DC/P	D	D	4.82	P	24.1	PD
34	42992646	c.209G > A	p.Arg70Gln	E	rs267607510	ENST00000586793.1	Head	VUS	D	DC/P	N	D	4.82	P	22.2	PD
35	42992641	c.214G > A	p.Glu72Lys	E	rs267607523	ENST00000586793.1	Head	P	D	DC	D	D	4.82	P	24	B
36	42992636	c.219G > C	p.Met73Ile	E	-	ENST00000586793.1	Coil1A	-	D	DC	N	D	4.82	P	23	B
37	42992636	c.219G > T	p.Met73Ile	E	-	ENST00000586793.1	Coil1A	-	D	DC	N	D	4.82	P	23	B
38	42992634	c.221 T > C	p.Met74Thr	E	rs267607504	ENST00000586793.1	Coil1A	P	D	DC	N	D	4.82	P	22.3	B
39	42992629	c.226C > T	p.Leu76Phe	E	rs57120761	ENST00000586793.1	Coil1A	P	D	DC	D	D	4.82	P	26.7	PD
40	42992624	c.231 T > A	p.Asn77Lys	E	-	ENST00000586793.1	Coil1A	-	D	DC	D	D	4.82	P	23.3	PD

No.	Position on Chromosome 17 (GRCh37)	HGVS DNA	HGVS protein	Exon/intron	SNP ID	Transcript	Coil	ClinVar	SIFT	MutationTaster	PROVEAN	FATHMM	GERP	ACMG	CADD	PolyPhen-2
41	42992623	c.232G > A	p.Asp78Asn	E	rs797044571	ENST00000586793.1	Coil1A	P	D	DC	D	D	4.82	P	26	PD
42	42992623	c.232G > C	p.Asp78His	E	-	ENST00000591880.1	Coil1A	-	D	DC	D	-	3.39	VUS/P	26	PD
43	42992621	c.234C > G	p.Asp78Glu	E	-	ENST00000586793.1	Coil1A	-	D	DC	D	D	4.82	P	26	PD
44	42992620	c.235C > T	p.Arg79Cys	E	rs59793293	ENST00000586793.1	Coil1A	P	D	DC	D	D	4.82	P	24.9	PD
45	42992619	c.236G > A	p.Arg79His	E	rs59285727	ENST00000586793.1	Coil1A	P	D	DC	D	D	4.82	P	24.6	PD
46	42992619	c.236G > C	p.Arg79Pro	E	rs59285727	ENST00000586793.1	Coil1A	P	D	DC	D	D	4.82	P	26.8	PD
47	42992619	c.236G > T	p.Arg79Leu	E	rs59285727	ENST00000586793.1	Coil1A	P	D	DC	D	D	4.82	P	26.7	B
48	42992605	c.250A > T	p.Ile84Phe	E	-	ENST00000587997.1	Coil1A	-	D	DC	D	D	5.07	LP	24.3	B
49	42992596	c.256_259delinsGAGT	p.Lys86_Val87delinsGluPhe	E	rs267607501	ENST00000586793.1	Coil1A	P	-	-	-	-		LP	-	-
50	42992596	c.259G > A	p.Val87Ile	E	rs267607518	ENST00000586793.1	Coil1A	P	D	DC	N	D	4.69	P	24	PD
51	42992593	c.262C > T	p.Arg88Cys	E	rs61622935	ENST00000586793.1	Coil1A	P	D	DC	D	D	4.69	P	28.2	PD
52	42992593	c.262C > A	p.Arg88Ser	E	rs61622935	ENST00000586793.1	Coil1A	P	D	DC	D	D	4.69	P	31	PD
53	42992577	c.278A > C	p.Gln93Pro	E	rs797044574	ENST00000586793.1	Coil1A	P	D	DC	D	D/T	4.69	LP	27.2	PD
54	42992553	c.302 T > C	p.Leu101Pro	E	rs267607516	ENST00000586793.1	Coil1A	P	D	DC	D	D	4.69	LP	24.3	PD
55	42992482	c.365_373dup	p.Arg124_Leu125insGlnLeuArg	E	rs797044575	ENST00000586793.1	Coil1B	P	−	−	−	−	−	LP	−	−
56	42992487	c.368 T > C	p.Leu123Pro	E	−	ENST00000586793.1	Coil1B	−	D	DC	D	D	4.69	LP	24.2	PD
57	42992470	c.380_385dupGCGGCT	p.Leu127_Asp128dup	E	−	ENST00000586793.1	Coil1B		−	−	−	−	−	LP	−	−
58	42992473	c.382G > A	p.Asp128Asn	E	rs267607509	ENST00000586793.1	Coil1B	P	D	DC	D	D/T	4.57	LP	24.2	PD
59	42991449	c.469G > A	p.Asp157Asn	E	rs59291670	ENST00000586793.1	Coil1B	B	D	DC	N	D/T	5.55	B	24.5	B
60	42992802	c.53G > T	p.Gly18Val	E	−	ENST00000586793.1	Head		T	P	N	D	3.25	VUS/P	1.67	B

No.	Position on Chromosome 17 (GRCh37)	HGVS DNA	HGVS protein	Exon/intron	SNP ID	Transcript	Coil	ClinVar	SIFT	MutationTaster	PROVEAN	FATHMM	GERP	ACMG	CADD	PolyPhen-2
61	42991103	c.611A > G	p.His204Arg	E	−	ENST00000586793.1	Coil1B	−	D	DC	D	D	4.71	LP	25	PD
62	42991101	c.613G > A	p.Glu205Lys	E	rs267607507	ENST00000586793.1	Coil1B	P	D	DC	D	D	4.71	LP	25.1	PD
63	42991097	c.617A > C	p.Glu206Ala	E	-	ENST00000586793.1	Coil1B	−	D	DC	D	D	4.71	P	33	PD
64	42990798	c.619G > A	p.Glu207Lys	E	rs267607500	ENST00000586793.1	Coil1B	P	D	DC	D	D	4.8	P	34	PD
65	42990798	c.619G > C	p.Glu207Gln	E	rs267607500	ENST00000586793.1	Coil1B	P	D	DC	D	D	4.8	P	33	PD
66	42990797	c.620A > T	p.Glu207Val	E	rs1555574517	ENST00000586793.1	Coil1B	LP	D	DC	D	D	4.8	P	32	PD
67	42990789	c.628G > A	p.Glu210Lys	E	rs57661783	ENST00000586793.1	Coil1B	P	D	DC	D	D	4.92	LP	31	PD
68	42990725	c.692 T > A	p.Leu231His	E	rs797044577	ENST00000586793.1	Coil2A	P	D	DC	D	D	4.92	LP	24.9	PD
69	42990713	c.704 T > C	p.Leu235Pro	E	rs60269890	ENST00000586793.1	Coil2A	P	D	DC	D	D	4.92	LP	24.9	PD
70	42990702	c.715C > G	p.Arg239Gly	E	rs58064122	ENST00000586793.1	Coil2A	VUS	D	DC	D	D	4.92	LP	25.3	PD
71	42990702	c.715C > T	p.Arg239Cys	E	rs58064122	ENST00000586793.1	Coil2A	P	D	DC	D	D	4.92	LP	25.3	PD
72	42990701	c.716G > A	p.Arg239His	E	rs59565950	ENST00000586793.1	Coil2A	P	D	DC	D	D	4.92	P	23.9	PD
73	42990693	c.724 T > A	p.Tyr242Asn	E	−	ENST00000586793.1	Coil2A	−	D	DC	D	D	4.92	LP	25	PD
74	42990686	c.731C > T	p.Ala244Val	E	rs61497286|	ENST00000586793.1	Coil2A	P	D	DC	N	D	4.94	LP	24.3	PD
75	42990678	c.739 T > C	p.Ser247Pro	E	rs267607519	ENST00000586793.1	Coil2A	P	D	DC/P	D	D	5.07	LP	23.1	PD
76	42990647	c.770A > G	p.Tyr257Cys	E	rs26760750	ENST00000586793.1	Coil2B	P	D	DC	D	D	5.07	LP	25.5	PD
77	42990639	c.778A > C	p.Lys260Gln	E	−	ENST00000586793.1	Coil2B	-	D	DC	D	D	5.07	LP	28.9	PD
78	42989147	c.799G > C	p.Ala267Pro	E	rs797044581	ENST00000586793.1	Coil2B	P	D	DC	D	D	4.42	LP	27.1	PD
79	42989143	c.803C > A	p.Ala268Asp	E	rs797044582	ENST00000586793.1	Coil2B	P	D	DC	D	D	4.42	LP	25.7	PD
80	42989137	c.809G > C	p.Arg270Pro	E	−	ENST00000586793.1	Coil2B	−	D	DC	D	D	4.42	LP	25.2	PD

No.	Position on Chromosome 17 (GRCh37)	HGVS DNA	HGVS protein	Exon/intron	SNP ID	Transcript	Coil	ClinVar	SIFT	MutationTaster	PROVEAN	FATHMM	GERP	ACMG	CADD	PolyPhen-2
81	42989119	c.827G > T	p.Arg276Leu	E	rs121909719	ENST00000586793.1	Coil2B	P	D	DC	D	D	4.42	LP	29.8	PD
82	42989078	c.868C > G	p.Gln290Glu	E	rs797044583	ENST00000586793.1	Coil2B	P	D	DC	D	D	4.38	LP	24.6	PD
83	42988797	c.934G > T	p.Glu312Ter		rs763868966	ENST00000586793.1	Coil2B	VUS	−	DC	−	−	4.65	P	22.8	−
84	42988743	c.988C > G	p.Arg330Gly	E	rs267607513	ENST00000586793.1	Coil2B	P	D	DC	D	D	4.51	LP	41	PD
85	42988737	c.994G > A	p.Glu332Lys	E	rs267607514	ENST00000586793.1	Coil2B	P	D	DC	D	D	4.51	LP	23.1	PD
86	42985511	c.1178G > T	p.Ser393Ile	E	rs62635764	ENST00000253408.5	Coil2B	P	T	DC	N	−	5.23	LP	24	B
87	42992483	c.372_373insGAA	p.Arg124_Leu125insGlu	E	-	ENST00000586793.1	Coil1B	-	-	-	−	−	4.63	LP	15.82	−
88	42990689	c.726_728dupAGG	p.E243dup	E	-	ENST00000586793.1	Coil1B	-	-	-	−	−	4.92	LP	16.67	−
89	42990716	c.701C > A	p.Ala234Asp	E	rs1353739896	ENST00000592320.1	Coil2A	-	T	DC/P	D	D	4.25	LP	19.72	PD
90	42990801	c.619-3C > G	NA	I	rs112611995	ENST00000586793.1	-	P	-	-	-	-	-	VUS/P	-	-
91	42992582	c.273A > C	p.Glu91Asp	E	-	ENST00000586793.1	Coil1A	-	D	DC	D	D	4.69	LP	25.6	PD
92	42992581	c.274C > G	p.Gln92Glu	E	-	ENST00000586793.1	Coil1A	-	D	DC	N	D	4.69	LP	24.9	PD
93	42992476	c.378_379dup	p.Leu127ArgfsTer26	E	-	ENST00000586793.1	Coil1B	-	-	-	-	-	4.58	P	17.26	-
94	42988612	c.1119G > C	p.Glu373Asp	E	-	ENST00000435360.2	Coil2B	-	D	DC	D	D	4.25	P	21.9	PD
95	42989044	c.902G > A	p.Gly301Asp	E	-	ENST00000586793.1	Coil2B	-	D	DC	D	D	4.38	LP	25.3	PD
96	42990644	c.773G > C	p.Arg258Pro	E	rs61726468	ENST00000586793.1	Coil2B	P	D	DC	D	D	5.07	LP	26	PD
97	42989155	c.791 T > C	p.Leu264Pro	E	rs797044579	ENST00000586793.1	Coil2B	P	D	DC	D	D	4.42	LP	25.3	PD
98	42992579	c.276G > T	p.Gln92His	E	-	ENST00000586793.1	Coil1A	-	D	DC	D	D	4.69	LP	24.5	PD

All the variants were analyzed based on the NM_002055, *D* damaging, *T* tolerated, *DC* disease causing, *B* benign, *P* polymorphism, *LP* likely pathogenic, *P* pathogenic, *PD* probably damaging, *VUS* variant of unknown significance, *N *neutral

## Results

Our genetic investigation revealed a novel de novo pathogenic variant, c.217A > G (p. Met73Val) in the recruited patient. Segregation analysis in the proband’s parents confirmed the identified variant of WES (Fig. 1B). The sequence alignments of proteins displayed the variant occurred within a highly conserved amino acid across various species, which provides its essential performance (Fig. 1C). Using schematic view of GFAP, the location of p.Met73Val was visualized. The identified variant is located on coil 1A of rod domain (Fig. 1D, E). Bioinformatic analysis by different tools such as Mutation Taster, PROVEAN, PolyPhen-2, CADD, SIFT, and GERP categorized this variant as disease causing, neutral (Score: -1.540), possibly damaging (Score: 0.526), PHRED: 21.8, damaging (Score: 0.005), and Score: 3.73, respectively.

**Fig. 2:**
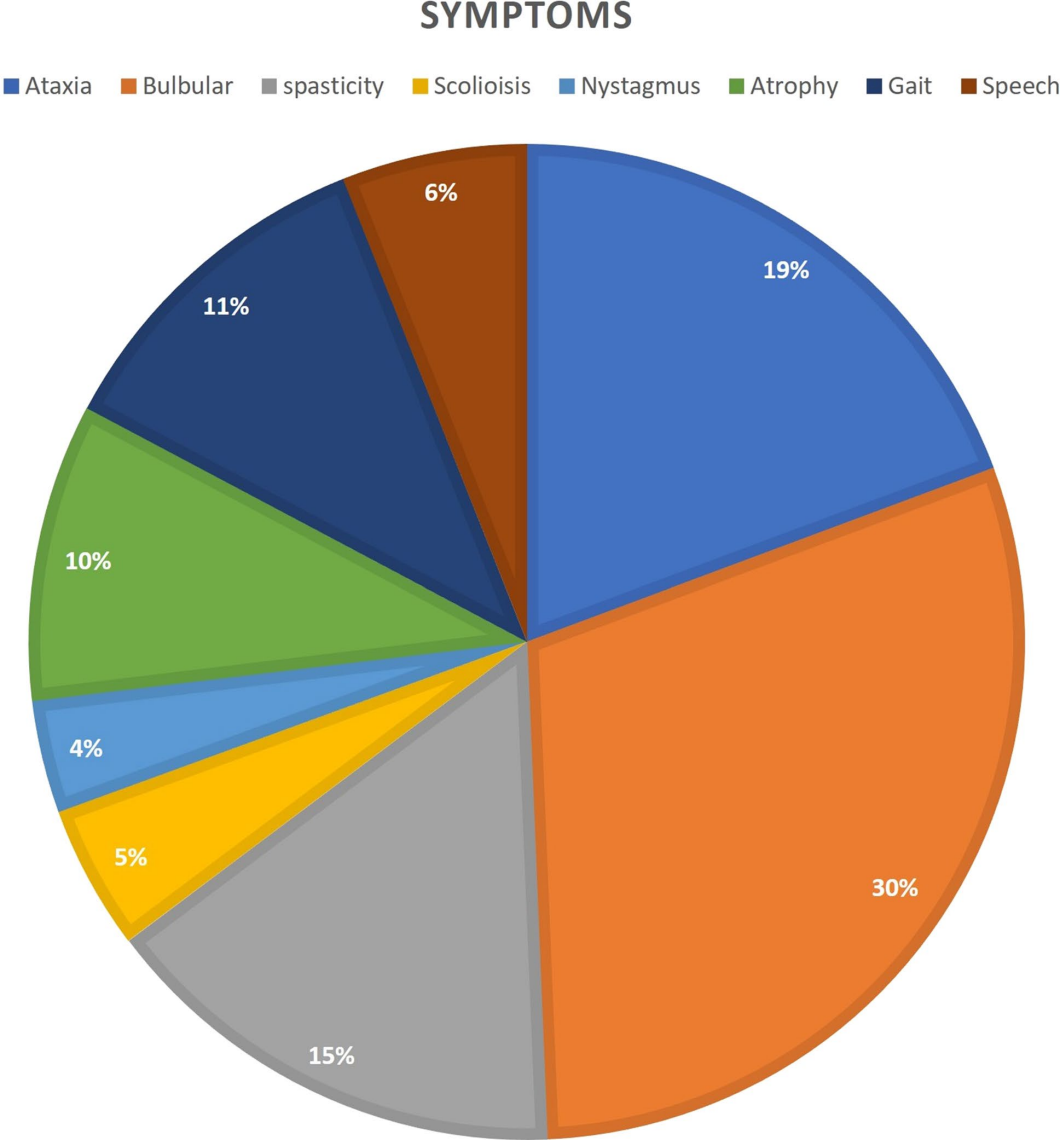
The clinical symptoms frequency among affected patients

Our search strategy and data extraction led to collection of 86 articles that met our defined inclusion criteria. Totally 377 patients were recruited in these articles, among them 212 patients were affected with juvenile or adult-onset form carrier of an alteration in *GFAP*. 202 mutations were reported and among them 98 were unique (without duplication). c.262C > T 11/212 (5.18%), c.1246C > T 9/212 (4.24%), c.827G > T 8/212 (3.77%), c.232G > A 6/212 (2.83%) were more frequent comparing to other fulfilled mutations. Our search analysis revealed that bulbar signs 115/212 (54.24%), ataxia 74/212 (34.9%) and spasticity 59/212 (27.83%) were the dominant clinical symptoms among carrier of *GFAP* variants (Fig. 2).

According to our analysis, mutations located on coil2B (24.74%) and coil1A (23.71%) constituted the majority of reported mutations in juvenile and adult-onset forms (Table 2). Among these 98 unique fulfilled variants 54 and 35 variants were categorized as likely pathogenic and pathogenic, respectively (Table 2).

## Discussion

Gain of function variants in *GFAP* are associated with different forms of AxD as a neurodegenerative disorder with autosomal dominant inheritance mode [3, 24]. GFAP is an important conserved intermediate filament protein with high expression level in astrocytes playing a significant role in central nervous system (CNS). Altered GFAP loses ability of extracellular K^+^ clearing and gliotic tissue hyperexcitability as the consequence [25]. This leads to astrocyte function impairment, demyelination changes and aggregation of Rosenthal fiber [26]. A comprehensive search on variants causing juvenile and adult was conducted and all the collected variants were analyzed by different in silico tools. Besides, our genetic analysis revealed a novel de novo variant in *GFAP* naming c.217A > G results in a methionine substitution to valine at codon 73 located in Coil 1A. GFAP-α (alpha) is the most abundant form of GFAP consists of head coil domain followed by the rod (filament) domain. Rod domain is also composed of four coils (1A, 1B, 2A, 2B). Reported variants near or within coil1A are Met73Lys, Met73Thr, and Met73Arg [13, 27–29]. Previous studies indicated that variants located within 1A, 1B and 2B domains may strongly cause severe form of AxD [13]. Met73Lys was first reported in a 7-month-old girl manifesting seizures and spasticity, but she did not indicate any bulbar signs or ataxia [27] and Met73Thr was reported in a 3-month-old girl. Her main clinical symptoms were macrocephaly, seizures, spasticity, bulbar signs, and ataxia [13]. Met73Arg is the third variant within this region and was reported in a patient with juvenile form. Her initial symptom was strabismus. In addition to the above-mentioned variants, Met73Ile and Met73Arg located in coil1A are also reported for patients affected with adult-onset form [30, 31]. Most of the reported mutations in GFAP gene are de novo and with 100% penetrance [3, 32]. A study conducted by Xiaoxuan Song et al. in 2021, two de novo mutations naming c.214G > A and c.1235C > T were reported in two unrelated individuals [33]. Both patients indicate regional neural activity increase. In this study, patient who was carrier of c.1235C > T manifests atrophy of grey matter mainly involving thalamus and bilateral putamen. Grey matter volume loss may be associated with disability in the long run [34]. AxD is inherited in autosomal dominant mode, however, in an investigation by Mu-Hui Fu et al.in 2020, a homozygous substitution naming c.197G > A (p.Arg66Gln) in a man with the onset age 16 was reported. This was the first report of a *GFAP* homozygous mutation [35].

Previous studies showed that c.715C > T (Arg239Cys) is the most common variant identified in Infantile AxD patients, however, c.262C > T (Arg88Cys) and c.1246C > T (Arg416Trp) are the two common variants of other two types. These variants are mainly located in Coil2B domain and Coil1A and therefore they are hotspot regions of *GFAP*. Our literature review indicated that bulbar signs, ataxia and spasticity constitutes the majority of clinical symptoms of *GFAP* carriers with juvenile and adult-onset AxD. A review conducted by Heshmatzad et al*.* in 2021 revealed that 59.70% of infantile AxD patients carrying a *GFAP* alteration, manifest seizure, spasticity, macrocephaly, and developmental as the dominant clinical symptoms [36]. These results indicated that spasticity is one of the most important signs among all AxD groups. Despite all the promising results of DNA analysis, next-generation sequencing [37] implementation, further studies are needed to categorize *GFAP* gene variants as a reliable genetic marker for AxD patients. There are only a few published articles investigating the genetics of Iranian patients affected with AxD [36, 38]. This fact highlights the important role of genetic in AxD diagnosis. More large-scale studies with the help of genetic analysis should be conducted in order to expand our knowledge of AxD.

## Accession Number

The accession number of the variant in ClinVar is as follows:

NM_002055.5 (GFAP): c.217A > G (p.Met73Val): VCV001173085.1.

## Data Availability

All data generated or analyzed during this study are included in this published article.
